# The Uremic Toxins Inorganic Phosphate, Indoxylsulphate, p-Cresylsulphate, and TMAO Induce the Generation of Sulphated Glycosaminoglycans in Aortic Tissue and Vascular Cells via pAKT Signaling: A Missing Link in the “Gut–Matrix Axis”

**DOI:** 10.3390/toxins17050217

**Published:** 2025-04-25

**Authors:** Christian Freise, Susanne Metzkow, Andreas Zappe, Monika Ebert, Nicola Stolzenburg, Julia Hahndorf, Jörg Schnorr, Kevin Pagel, Matthias Taupitz

**Affiliations:** 1 Department of Radiology, Campus Mitte, Charité—Universitätsmedizin Berlin, Corporate Member of Freie Universität Berlin and Humboldt-Universität zu Berlin, 10117 Berlin, Germanymatthias.taupitz@charite.de (M.T.); 2 Department of Biology, Chemistry and Pharmacy, Freie Universität Berlin, 14195 Berlin, Germany

**Keywords:** uremic toxins, inorganic phosphate, glycosaminoglycans, chronic kidney disease, vascular smooth muscle cells, aortic rings, chondroitin sulphate, heparan sulphate, hyaluronic acid, gut–matrix axis, sulphated glycosaminoglycans

## Abstract

Gut-derived uremic toxins (UTs) contribute to cardiovascular disorders like atherosclerosis and cardiomyopathy in patients with chronic kidney disease (CKD), causing increased cardiovascular morbidity and mortality. The intermediate steps between higher concentrations of gut-derived UTs and organ damage caused by UTs are still insufficiently understood. Glycosaminoglycans (GAGs) as components of the extracellular matrix are known to interact with various ligands such as growth factors or receptors, thereby influencing (patho)physiological processes. We previously found that the UT inorganic phosphate (Pi) induces the synthesis and sulphation of the GAGs heparan sulphate and chondroitin sulphate in the rat vascular smooth muscle cell (VSMC) line A7r5 and in the human endothelial cell (EC) line EA.Hy926. The aim of this study was to investigate if other organic UTs modulate GAGs in vascular cells as well. We treated ex vivo cultures of rat aortic rings as well as primary rat VSMCs and human ECs with the UTs Pi, indoxylsulphate (IS), p-cresylsulphate (pCS), trimethylamine N-oxide (TMAO), and urea, and analyzed the samples by histological staining, qPCR, western blot, HPLC, and colorimetric assays. The UT treatment of aortic rings and cells increased contents of sulphated GAGs and hyaluronic acid. UT-treated cells contained higher amounts of 4S- and 6S-sulphated GAGs compared to controls. This was accompanied by altered expressions of genes and proteins relevant for GAG metabolism. Mechanistically, the effects of the UTs on GAGs involve the activation of the PI3K/Akt pathway and of the transcription factor NF-κB. In conclusion, the UT-induced remodeling of the cardiovascular matrix by upregulation of sulphated GAGs and hyaluronic acid in aortic tissue and vascular cells might be a missing link between gut-derived UT and pathophysiological alterations in the cardiovascular system in the sense of a gut–matrix axis.

## 1. Introduction

Uremic toxins (UTs) are metabolites that are generated from food by bacteria in the gut before being absorbed by bloodstream. UTs that are still present as precursor molecules are converted to mature UTs in the liver, e.g., by enzyme-mediated hydroxylation [1]. UTs are usually cleared by the kidneys. During chronic kidney disease (CKD), they are retained in the body, contribute to the progression of CKD, and have pathophysiological effects on various organ systems, including the cardiovascular system [2,3].

For instance, the organic UTs indoxyl sulphate (IS) and para-cresyl sulphate (pCS) contribute to endothelial dysfunction and vascular damage [4,5]. Further, circulating levels of trimethylamine N-oxide (TMAO) promote vascular calcification and inflammatory signaling in rats with CKD [6]. Also, the UT inorganic phosphate (Pi) is linked to vascular calcification and a high risk of cardiovascular events and mortality [7,8].

Besides a reduced clearance, the generation of UTs is increased during CKD by an altered gut microbiota [9,10]. In chronic dialysis patients, an increased gut wall inflammation is observed and in CKD animals, a colon wall inflammation has been shown to be associated with a disruption of the epithelial tight junction barrier and subsequent translocation of bacterial DNA and endotoxins into the bloodstream [11,12]. This “leaky gut” therefore also contributes to an enhanced systemic exposure with UTs [13,14,15] and subsequent pathophysiological effects on different organ systems which led to the emergence of several terms describing “gut–organ” axes like the gut–liver axis or the gut–bone axis [16]. However, intermediate steps between high concentrations of gut-derived UTs and pathophysiological effects on various organ systems are still insufficiently understood.

We previously found that a remodeling of the cardiovascular extracellular matrix (ECM) by UTs might be a missing puzzle piece. Amongst others, we found that UTs stimulate the generation and modify the composition of exosome-like extracellular vesicles from rat vascular smooth muscle cells (VSMCs) and human endothelial cells (ECs) [17,18,19]. Besides affecting the miRNA cargo load of the extracellular vesicles, a mixture of urea and IS and especially Pi also increased the contents of the glycosaminoglycans (GAGs) heparan sulphate (HS) and chondroitin sulphate (CS) in the vesicles and influenced their degree of sulphation [17,18,19].

Together with HS and CS, the GAGs dermatan sulphate, heparin, keratan sulphate [20], and hyaluronic acid (HA) [21] are linear polysaccharides which consist of repeating disaccharide units of uronic acid and hexosamine [22]. An exception is keratan sulphate, which consists of a sulphated poly-N-acetyl lactosamine chain made of d-galactose and N-acetylglucosamine instead of uronic acid [20]. Except for HA, all GAGs are sulphated to different degrees and at different positions and are covalently bound to proteins to form proteoglycans. These different degrees of GAG sulphation and the different possible sulphation patterns provide a very variable structural diversity of the GAGs [23,24]. This makes GAGs important modulators for the interaction of cells and extracellular vesicles with ligands such as receptors, growth factors, and other components of the ECM [17,23,24,25,26,27].

The latter, and the increasingly important role of UTs for (patho)physiological processes [28], therefore led to the aim of the present study: to investigate the effects of UTs on the abundance and sulphation of GAGs in rat aortic tissues ex vivo, in isolated primary VSMCs from rats and human ECs. We expect this to lead to new insights regarding the link between elevated concentrations of UTs and an ECM-specific remodeling of the cardiovascular system in the sense of a “gut–matrix axis” as a starting point for subsequent pathophysiological alterations.

## 2. Results

### 2.1. UTs Increase Contents of Sulphated GAGs (sGAGs) in Rat Aortic Ring Cultures

**Figure 1:**
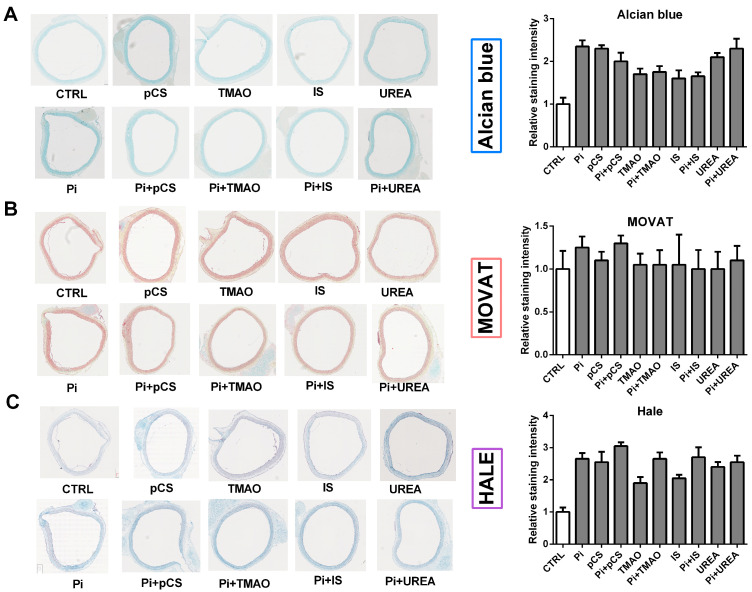
Histological staining of sGAGs in aortic ring cultures. Aortic rings from rats were treated for 7 d with 3.0 mM NaH_2_PO_4_ (Pi, final concentration), 50 µg/mL IS, 100 µM TMAO, 20 mM UREA, and 100 µg/mL pCS. The sGAGs were visualized by (**A**) alcian blue staining, (**B**) MOVAT pentachrome staining, and (**C**) HALE staining. Staining intensities were quantified using ImageJ software (Version 1.50i). Shown are representative pictures of two replicates from two independent experiments with corresponding quantification graphs. Abbr.: 5-UT, mixture of the five individual uremic toxins; EC, endothelial cells; sGAGs, sulphated glycosaminoglycans; IS, indoxyl sulphate; pCS, p-cresylsulphate; Pi, inorganic phosphate; TMAO, trimethylamine N-oxide.

The first aim was to test whether treatment with UTs increases contents of sGAGs in aortic ring cultures. We also aimed to optimize the visualization of sGAGs in aortic tissues. The classical alcian blue staining showed slightly more intense, blue-stained sGAG-rich areas in tissues treated with UTs compared to the control. Treatments with Pi and pCS had the strongest effects on the abundance of sGAGs in the tissues (Figure 1A). Of note, the effects of a simultaneous treatment with both UTs were slightly weaker than the effects of the individual UTs.

In parallel, we applied the MOVAT pentachrome staining method. This staining method showed no distinct differences between treatment groups and the control group (Figure 1B). The HALE staining method enabled the detection of more pronounced differences within the treatment groups and in comparison to the control (Figure 1C). The treatments with Pi, pCS, and a combination of both showed the strongest effects (Figure 1C).

### 2.2. UTs Modify the Expression of GAG-Specific Genes and Increase Contents of sGAGs in Aortic Tissues

We next investigated if the UT-induced sGAG contents in the aortic rings were reflected by an altered expression of genes which encode for proteins being involved in the generation, the sulphation, the desulphation, and the breakdown of GAGs. Due to the limited number of samples, we compared only effects of Pi alone and a mixture of the five UTs (5-UT) with respective controls.

**Figure 2:**
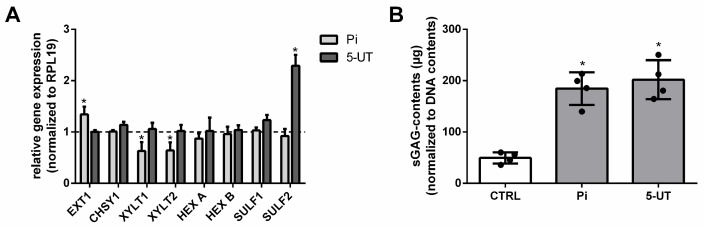
Pi and 5-UT modify the expression of GAG-specific genes and increase the contents of sGAGs in aortic tissues. (**A**) Aortic ring cultures were treated for 7d with Pi, 5-UT, or vehicles. Gene expressions were determined by qPCR and normalized to ribosomal protein L19 (RPL19) expressions. * *p* < 0.05 versus control (*n* = 3). (**B**) Treatment-dependent effects on sGAG contents in the cells were determined using the BlyscanTM assay. Shown are means ± SD (n = 3, * *p* < 0.05). Abbr.: 5-UT, mixture of the five individual uremic toxins; sGAGs, sulphated glycosaminoglycans; Pi, inorganic phosphate.

Pi induced a significant upregulation of EXT1 and a significant downregulation of XYLT1 and XYLT2 (Figure 2A). The UT mixture 5-UT did not induce distinct differences in gene expression levels compared to control except for the gene SULF2, which was significantly upregulated (Figure 2A).

In parallel, we applied tissue samples to the Blyscan^TM^-based analyses of sGAGs. Figure 2B shows that Pi and 5-UT significantly increased levels of sGAGs in the aortic tissue samples compared to the control.

### 2.3. UTs Increase Contents of sGAGs Also in Vascular Smooth Muscle Cells and Endothelial Cells

To complement the results from the aortic tissues, we also analyzed the effects of the UTs on the generation of sGAGs in cultures of VSMCs and ECs in vitro.

**Figure 3:**
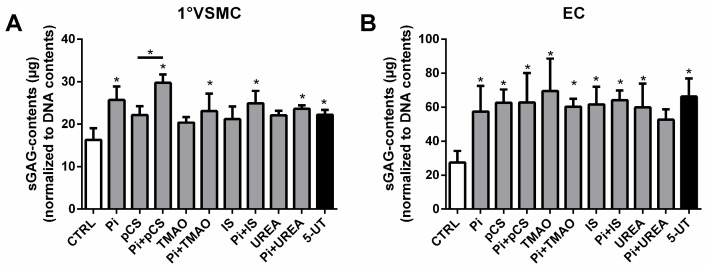
UTs increase sGAG contents in vascular cells in vitro. (**A**) Primary rat VSMCs and (**B**) human ECs were treated with UT as indicated for 7 d. The sGAG contents in cells were quantified using the Blyscan^TM^ assay (Biocolor, Carrickfergus, UK). Shown are means ± SD (*n* = 3, * *p* < 0.05). Abbr.: 5-UT, mixture of the five individual uremic toxins; ECs, endothelial cells; sGAGs, sulphated glycosaminoglycans; IS, indoxyl sulphate; pCS, p-cresylsulphate; Pi, inorganic phosphate; TMAO, trimethylamine N-oxide; VSMCs, vascular smooth muscle cells.

The experiments revealed similar effects of UTs on sGAGs in both cell lines as in the aortic tissue experiments. In the primary VSMC, treatment with Pi alone and in respective combinations with the other UTs led to significantly elevated sGAG contents. The strongest effects were observed for a mixture of Pi with pCS, and 5-UT induced significantly higher sGAG contents compared to control as well (Figure 3A).

For the EC, almost every treatment with individual toxins or a respective combination with Pi significantly elevated sGAG contents in the cells (Figure 3B). No distinct differences among the treatment groups were observed. In direct comparison between VSMCs and ECs, the ECs tend to react more sensitively to the UT treatment. This is indicated by an up to ~3-fold increase in sGAG contents in ECs compared to an only ~2-fold increase in VSMCs (Figure 3A,B).

### 2.4. UTs Regulate GAG-Specific Genes and Proteins in Vascular Cells

Like in the aortic tissues, we also observed the effects of the UTs on GAG-relevant genes in primary VSMC. Since diabetes is the most common cause of kidney failure worldwide, we also applied glucose as a treatment group.

**Figure 4:**
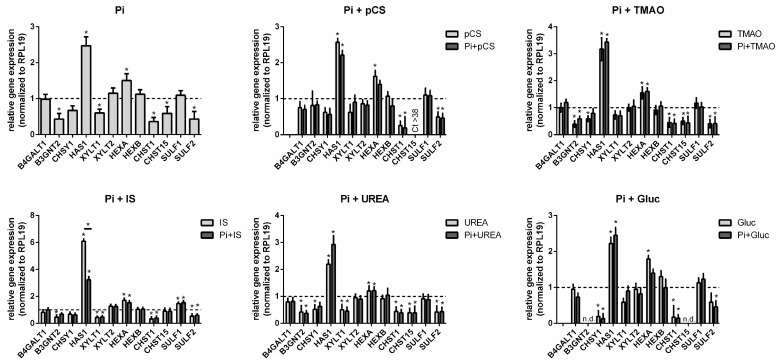
UTs influence the expression of GAG-relevant genes and proteins in primary rat VSMCs. Primary rat VSMCs were treated with UTs for 7 d as indicated. Gene expressions were determined by qPCR and normalized to RPL19 expressions. Shown are means ± SD (*n* = 3, * *p* < 0.05). Abbr.: Gluc, glucose; IS, indoxyl sulphate; pCS, p-cresylsulphate; Pi, inorganic phosphate; TMAO, trimethylamine N-oxide; VSMCs, vascular smooth muscle cells.

What was striking was that all treatments resulted in a significant increase in the gene expression of HAS1 and HEXA in the cells. In addition, all tested toxins induced a significant downregulation of B3GNT2, CHSY1, XYLT1, CHST1, and SULF2 expression (Figure 4). A co-treatment of the toxins with Pi provoked no distinct changes in gene expressions except for IS, where the presence of Pi significantly reduced the gene expression of HAS1 (Figure 4).

**Figure 5:**
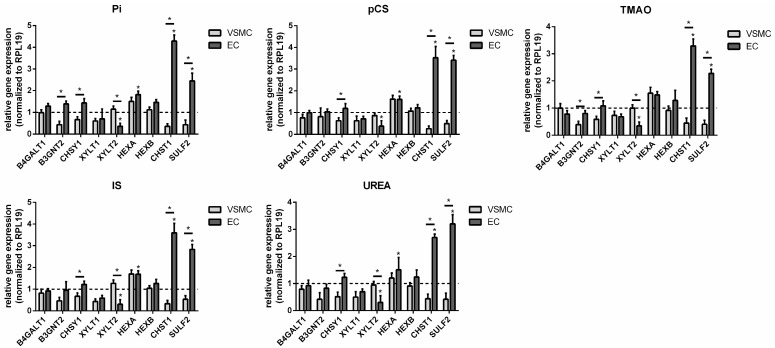
Comparison of the UT-mediated effects on GAG-relevant gene expressions in VSMCs and ECs. The vascular cells were treated with UTs for 7 d as indicated. Gene expressions were determined by qPCR and normalized to RPL19 expressions. Shown are means ± SD (*n* = 3, * *p* < 0.05). Abbr.: ECs, endothelial cells; IS, indoxyl sulphate; pCS, p-cresylsulphate; Pi, inorganic phosphate; TMAO, trimethylamine N-oxide; VSMCs, primary rat vascular smooth muscle cells.

We additionally tested the effects of the individual toxins in ECs and compared them with the effects in the VSMCs. A common feature between both cell types is a UT-induced upregulation of HEXA (Figure 5). However, we also observed significant differences between both cell lines. Contrary to VSMCs, the expression of genes B4GALT1, B3GNT2, and CHSY1 were not downregulated but were even partially increased in ECs (Figure 5). A further distinct difference is a strong upregulation of CHST1 and SULF2 in ECs (Figure 5). We did not include data for HAS1 and SULF1 gene expressions in ECs, since the respective Ct values were in the range between 38 and 40.

**Figure 6:**
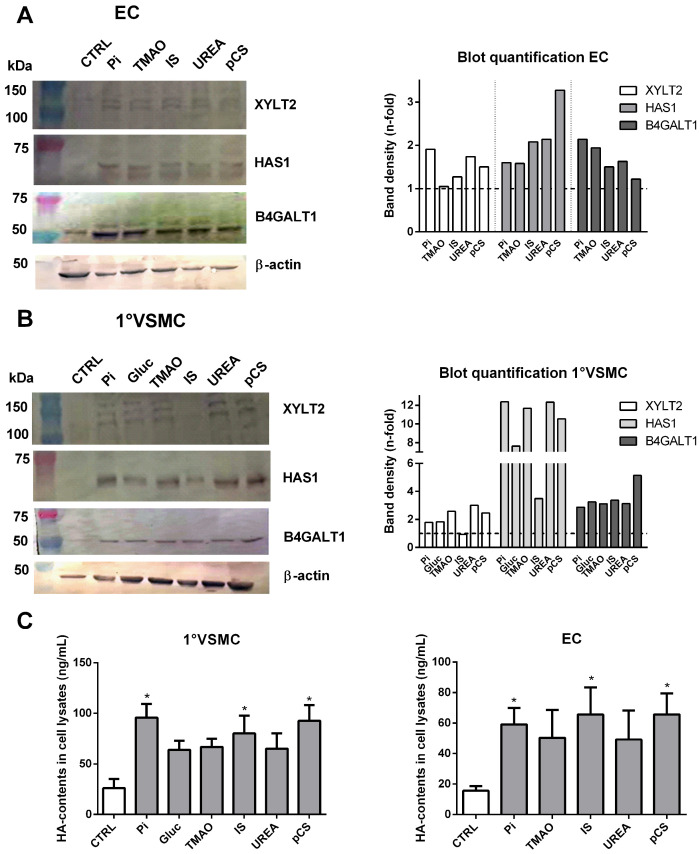
UTs induce expressions of proteins to be involved in the biosynthesis of GAGs. (**A**) ECs or (**B**) primary VSMCs were treated with UTs as indicated for 7 d. Protein expressions were determined by western blot. Shown are representative blots of three independent experiments along with graphs from quantification of respective band intensities. (**C**) ECs and VSMCs were treated with UTs for 7 d as indicated. HA contents in lysed cells were analyzed by ELISA (means ± SD, *n* = 3). * *p* < 0.05. Abbr.: 1°VSMCs, primary rat vascular smooth muscle cells; ECs, human endothelial cells; Gluc, glucose; HA, hyaluronic acid; IS, indoxylsulphate; pCS, p-cresylsulphate; Pi, inorganic phosphate; TMAO, trimethylamine N-oxide.

To complement the qPCR measurements, we determined UT-mediated effects on protein expressions of XYLT2, HAS1, and B4GALT1. All three proteins are involved in the cellular biosynthesis of GAGs. All tested UTs induced a distinct upregulation of these proteins in ECs compared to control (Figure 6A). In VSMCs, we observed similar inducing effects of the UTs on the proteins, including that of Gluc (Figure 6B). The only exception was the effect of IS on the protein expression of XYLT2, which showed no differences compared to the control (Figure 6B).

The stimulating effects of the UTs on protein expressions of HAS1 led to additional analyses of HA contents in lysates of UT-treated VSMCs and ECs. All individual UTs increased HA contents of analyzed cell lysates (Figure 6C). However, in both cell types, significant effects were seen only after treatments with Pi, IS, and pCS (Figure 6C). Of note, glucose treatment of VSMCs slightly increased HA contents as well (Figure 6C).

### 2.5. Treatment of Vascular Cells with UTs Influences the Degree of Sulphation of Cellular GAGs

**Figure 7:**
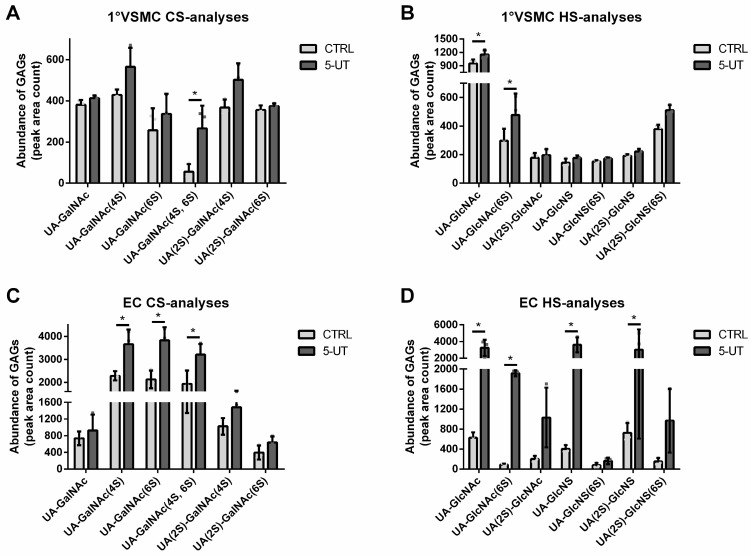
UTs induce changes in sulphate patterns of GAGs in vascular cells. (**A**,**B**) Primary VSMCs and (**C**,**D**) ECs were treated with 5-UT for 7 d or left untreated. Analyses of sulphate patterns in GAG oligosaccharides were performed by HPLC. Shown are means ± SD (*n* = 3, * *p* < 0.05). Abbr.: 1°VSMCs, primary rat vascular smooth muscle cells; 5-UT, mixture of the five uremic toxins inorganic phosphate, indoxylsulphate, p-cresylsulphate, trimethylamine N-oxide and urea; ECs, human endothelial cells, GAG, glycosaminoglycans.

Since UTs influenced the expressions of genes being involved in the sulphation and desulphation of GAGs, we analyzed treatment-dependent changes in the structure and the degree of GAG sulphation in both cell types. Primary VSMCs treated with 5-UT contained slightly increased overall levels of HS and CS compared to control (Figure 7A,B). In addition, the UT-treated cells contained significantly more 4-O-/6-O-sulphated CS (Figure 7A) and 6-O-sulphated HS compared to control (Figure 7B).

For the ECs, the effects of the UTs were even more pronounced. The UT-treated cells contained significantly higher levels of 4-O- and 6-O-sulphated CS (Figure 7C). Further, the contents of HS oligosaccharides were markedly increased in 5-UT-treated EC, including significantly higher contents of 6-O-sulphated UA-GlcNAc (Figure 7D).

### 2.6. The GAG-Specific Effects of the UTs in Vascular Cells Involve Activation of the PI3K/AKT Pathway and Activation of NF-κB Signaling

We recently found that treatment of vascular cells with Pi goes along with enhanced PI3K/AKT signaling [18]. We expanded these studies by also testing the effects of 5-UT and Pi in primary VSMCs and ECs.

**Figure 8:**
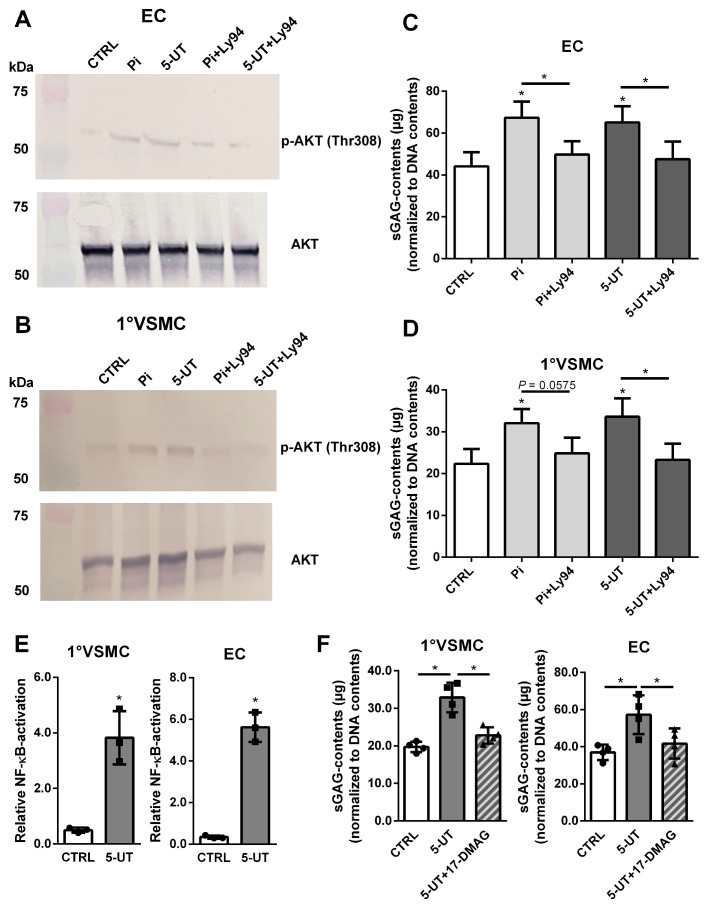
The UT-mediated effects on sGAG contents in vascular cells involve PI3K/AKT signaling and activation of NF-κB. (**A**) Primary VSMCs and (**B**) ECs were treated with Pi or 5-UT for 15 min ± the PI3K/AKT-inhibitor Ly294002. Protein expressions and phosphorylation of AKT were determined by western blot. Shown are representative blots of three independent experiments. (**C**,**D**) Primary VSMCs (**C**) and ECs (**D**) were treated 3 d with Pi or 5-UT ± the PI3K/AKT-inhibitor Ly294002. Treatment-dependent effects on sGAG contents in the cells were determined using the Blyscan^TM^ assay. Shown are means ± SD (*n* = 3, * *p* < 0.05). (**E**) Primary VSMCs and ECs were treated 24 h with 5-UT. Treatment-dependent effects on activation of NF-κB were determined by a luminescent luciferase reporter assay. Shown are means ± SD (*n* = 3, * *p* < 0.05). (**F**) Cells were treated for 3 d as indicated. Effects of the NF-κB-inhibitor 17-DMAG on 5-UT-induced sGAG contents in vascular cells were determined using the Blyscan^TM^ assay. Shown are means ± SD (*n* = 3, * *p* < 0.05). Abbr.: 17-DMAG, inhibitor of NF-κB; 5-UT, mixture of the five uremic toxins inorganic phosphate, indoxylsulphate, p-cresylsulphate, trimethylamine N-oxide and urea; ECs, endothelial cells, GAPDH, glyceraldehyde 3-phosphate dehydrogenase; Ly29, Ly294002—PI3K/AKT-inhibitor; Pi, inorganic phosphate; sGAGs, sulphated glycosaminoglycans; VSMCs, vascular smooth muscle cells.

As expected, a treatment with Pi and with 5-UT provoked a phosphorylation of AKT in primary VSMCs (Figure 8A) and ECs (Figure 8B), which could be blocked by the PI3K/AKT-inhibitor Ly294002. We consequently checked if Ly294002 interferes with the UT-induced increase in sGAGs in both cell types. Indeed, a blockade of PI3K/AKT signaling blocks the UT-mediated stimulation of sGAG contents in both ECs (Figure 8C) and VSMCs (Figure 8D).

In addition, we also investigated the effects of UTs on the activation of the transcription factor NF-κB (nuclear factor kappa-light-chain-enhancer of activated B cells). Figure 8E shows that treatment with 5-UT for 24 h induced the activation of NF-κB. Like the inhibition of PI3K/AKT, the inhibition of NF-κB by the inhibitor 17-DMAG also blocked the 5-UT-induced increase in sGAG contents in both cell lines (Figure 8F).

### 2.7. In Vivo-Derived Aortic Rings from CKD Rats Contain More sGAGs Compared to Healthy Controls

**Figure 9:**
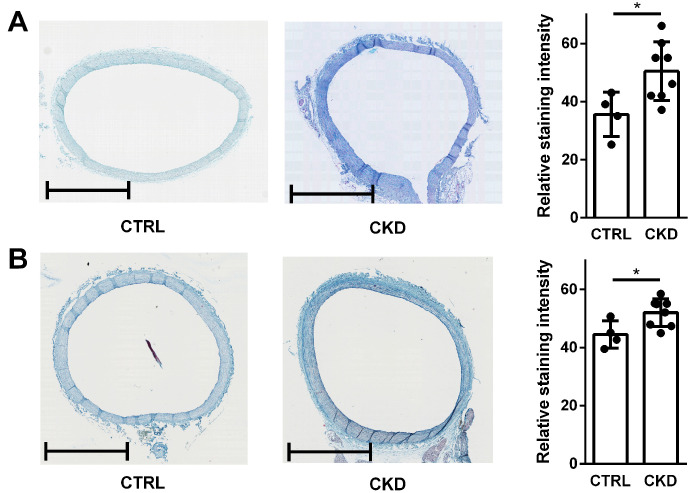
Histological staining of sGAGs in in vivo-derived aortic rings. Aortic rings were isolated from rats with CKD or healthy controls. Sulphated GAGs were visualized by (**A**) alcian blue staining or by (**B**) HALE staining. Shown are representative pictures of two replicates from *n* = 8 animals (CKD group) or *n* = 4 animals (control group) along with the corresponding analyses of staining intensities (* *p* < 0.05). Bars = 1 mm.

We finally studied if our experimental approach in vitro/ex vivo could in principle be translated to the in vivo situation. Indeed, alcian blue staining (Figure 9A) and HALE staining (Figure 9B) of sGAGs in the aortic rings isolated from rats with CKD is distinctly more intense than in aortas from healthy control animals.

## 3. Discussion

We here show that the UTs IS, pCS, TMAO, urea, and Pi increase sGAG contents in rat aortic tissues as well as in rat-derived primary VSMCs and in the human EC cell line EA.Hy926. This goes along with an increased degree of sulphation of the GAG oligosaccharides heparan sulphate and chondroitin sulphate. Our data indicate that the UT-mediated increase in sGAG contents is due to the induction of PI3K/AKT signaling and activation of the transcription factor NF-κB. Complementing previous studies [18,19], this supports the hypothesis that the interplay between gut-derived UTs and GAGs is an important component of the cardiovascular remodeling during CKD and, thus, might be referred as a “gut–ECM axis”.

We previously demonstrated that Pi induces elevated contents of sGAGs in the rat VSMC cell line A7r5 and in the human endothelial cell line EA.Hy926 [18,19]. However, these studies placed an emphasis on the analysis of isolated exosome-like extracellular vesicles from the cells. This included analyses of GAG oligosaccharides in the vesicles and consequences for their interaction with ligands.

In the present study, we focused on the analysis of GAG-specific effects of Pi as an inorganic UT and additional organic UTs on a cellular level, including rat aortic tissue cultures, primary rat-derived VSMCs, and human ECs. The first challenge was the visualization of UT-induced sGAGs in the ex vivo cultures of the aortic rings. To avoid time-consuming and expensive methods such as mass spectroscopy or using specific antibodies available for a few GAGs only, we applied three different histological staining methods as a semi-quantitative alternative.

The alcian blue staining technique [29] is based on polyvalent, copper-containing basic dyes that react with sulphated and carboxylated GAGs [30]. This method enabled us to detect distinct differences between control and treatment groups. However, differences between the treatment groups were barely visible. A second alternative therefore was the MOVAT staining method, which covers five stains: alcian blue, crocein scarlet, and Verhoeff hematoxylin, along with saffron and acidic fuchsine [31]. This allows simultaneous staining of collagen, elastin, mucin, muscle, and fibrin in tissue sections. However, we did not observe distinct differences between control and treatment groups. A third alternative was HALE staining [32]. In contrast to the other two methods, it is based on the reaction of cationic colloidal iron with carboxyl and sulphate residues, which also results in a blue staining of sGAGs. Like alcian blue, HALE staining also proved to be suitable for visualization of clear differences between the control and treatment groups.

Regardless of the differences in staining methods, all three indicated that a treatment with UTs increased contents of sGAGs in the aortic tissue.

Subsequent analyses of the aortic ring cultures revealed some effects of UTs on GAG-relevant gene expressions. Pi induced a weak but significant upregulation of the gene EXT1, which synthesizes HS chains [33]. Interestingly, Pi also induced a simultaneous downregulation of the genes XYLT1 and XYLT2, which both contribute to the biosynthesis of GAG chains [34]. In contrast, a treatment with 5-UT provoked no effects on these genes but significantly elevated the expression of SULF2, which encodes for a sulfatase that removes 6-O-sulphate groups from heparan sulphate [35]. Of note, the data only represent a snapshot after 7 days of treatment and further studies might require more focused measurements e.g., qPCRs at different timepoints. However, the gene and the protein measurements clearly point to the fact that UTs influence the turnover of GAGs in the tissue.

The data are consistent with previous results which demonstrate that arteries of uremic rats contained significantly increased contents of sGAGs compared to controls [36]. However, most previous studies on the relationship between CKD or uremic conditions/UTs and arterial remodeling have focused predominantly on endothelial dysfunction, altered cellular functions of VSMCs, and increased arterial stiffness [37,38,39].

The present study on the remodeling of GAGs therefore adds new puzzle pieces to the understanding of the relationship between CKD, increased plasma levels of UTs, and a cardiovascular remodeling of the ECM. We further investigated the interplay between UTs and sGAGs using individual vascular cells in vitro. In primary rat VSMCs, foremost Pi and 5-UT induced the abundance of sGAGs on protein level. In ECs, however, the individual UTs increased the sGAG contents. This indicates different sensitivities of VSMCs and ECs to the UTs and might be one factor contributing to the different responses of aortic tissue and individual cells to the treatment with UTs.

Subsequent qPCR measurements confirmed this assumption in that those genes being responsible for the synthesis of GAGs like B3GNT2, CHSY1, or XYLT1,2 [34,40,41] were differently regulated by UTs in both cell types. The most striking difference was the strong upregulation of CHST1 and SULF2 in ECs compared to VSMCs. These genes encode for a sulphotransferase [42] and a sulphatase [35], respectively, and therefore could impact the structure and the degree of sulphation of cellular GAGs. This was reflected by analyses of the contents of CS and HS oligosaccharides in the cells. Predominantly, the ECs contained significantly more sulphated residues of CS and HS due to UT treatment, while in VSMCs, only a moderate increase was observed.

On protein level, we confirmed through examples that, indeed, some of the analyzed genes were finally translated into proteins. Both cell types contained distinctly more protein contents of XYLT2, HAS1, and B4GALT1 when treated with UTs. This explains the observed increase in sGAG contents in UT-treated cells, including elevated contents of HA. An interesting finding was that we detected HAS1 expression and HA contents in ECs on protein level but found only minor amounts of HAS1 gene expression in the cells (Ct values ~40) after equal times of UT treatment. This points to a time-limited gene expression of HAS1 and/or the involvement of other HA-synthesizing enzymes like HAS2 [43], which should be considered in follow-up studies.

The regulation of GAGs by pathophysiological stimuli has also been demonstrated in other studies. For instance, an altered synthesis and sulphation of GAGs has been demonstrated in a mice model of dextran sulphate sodium-induced colitis [44]. Also, TGF-β was shown to induce CHSY1 expression in VSMCs [45], while TNF-α induces B4GALT1 in HUVECs [46] and was also shown to induce the expression of HA on cultured EC lines and primary endothelial cultures [47]. Our group also recently demonstrated stimulatory effects of Pi and of a mixture of urea and IS on contents of sGAGs in vascular cell-derived exosome-like extracellular vesicles [18,19].

Diabetes is the most common cause of kidney failure worldwide, and high glucose levels are known to promote VSMC dysfunction by stimulating inflammatory gene expression, migration, and proliferation [48,49,50]. Indeed, when applied as one treatment group in gene and protein expression studies with VSMCs, we found that glucose regulated GAG-specific gene and protein expressions in VSMCs. This included the induction of higher contents of HA in the cells, which confirmed data of a previous study [43]. Our results suggest that glucose might also contribute to the remodeling of GAGs in the cardiovascular system. However, this requires more focused studies in the future.

Aiming to understand the underlying mechanisms of the UT-mediated regulation of GAGs in vascular cells, we finally tested the effects of the UTs on the activation of the PI3K/Akt signaling pathway. This pathway is involved in the remodeling of the ECM, including ECs and VSMCs [51,52]. We recently found that Pi and a mixture of urea and IS stimulated PI3K/AKT signaling and expression of the sulphate transporter SLC26A2 in the rat VSMC cell line A7r5 and in the EC line EA.Hy926, which leads to elevated contents of GAGs in the cells and isolated exosome-like extracellular vesicles thereof [17]. The present study complemented these previous data by showing that Pi and 5-UT induced PI3K/AKT signaling also in primary rat VSMCs and ECs. This leads to increasing sGAG contents in the cells, as implicated by the fact that these effects of the UTs could be blocked by the PI3K/AKT-signaling inhibitor LY294002. This suggests further studies on therapeutic interventions.

Other studies also describe the effects of UTs on PI3K/AKT signaling. IS was shown to stimulate calcification of human VSMC via PI3K/AKT signaling [53] and TMAO could promote autophagy inhibition in VSMC in atherosclerosis [54]. For pCS, effects on PI3K/AKT signaling have so far only been described in other cell types like renal fibroblasts [55] or prostate cancer cell lines [56].

Another component of cellular signaling in CKD is the activation of NF-κB. NF-κB plays a significant role in the progression of CKD by regulating genes involved in inflammation, immune responses, and cell survival [57,58]. Interestingly, both excessive activation and partial inactivation of NF-κB signaling can lead to adverse effects, such as hypertension and kidney dysfunction [58]. Additionally, targeting specific pathways, like the non-canonical NF-κB signaling, has shown promise in reducing inflammation and fibrosis in CKD models [59]. This fits with our data, where the inhibition of NF-κB also inhibits toxin-induced sGAG production in vascular cells.

However, a comprehensive identification of relevant signaling pathways influenced by the toxins requires significantly more extensive analyses in follow-up studies, preferably with human sample material.

The final examination of in vivo-derived aortic tissues complemented our in vitro/ex vivo data. CKD-derived aortas indeed contained more sGAGs than control tissues. Without wanting to anticipate further study results, this points to the possibility of translating our conclusions to the in vivo situation and provides a rationale for further investigations in animal models of CKD.

In summary, the data of this study point to UTs being regulators of the ECM in cardiovascular tissues/cells. These ECM alterations, in turn, might favor the development of further pathophysiological changes in the cardiovascular system. Together with the fact that a disruption of the epithelial tight junction barrier during CKD (leaky gut) augments plasma levels of UTs [13,14], UTs could be considered as mediators within a “gut–matrix axis”.

## 4. Limitations

Our in vitro/ex vivo experiments were performed under static culture conditions. However, a recent study showed that flow conditions can influence cell surface structures like the glycocalyx [60].

## 5. Conclusions

The results might contribute to a better understanding of UT-involved pathophysiological processes in the cardiovascular system during CKD. So far, the link between high concentrations of UTs and their pathophysiological effects on organs is insufficiently understood. GAGs are known to be involved in the regulation of many (patho)physiological processes. In addition to the simple number of GAGs, their degree of sulphation also plays a role. The fact that gut-derived UTs increase the amount and the sulphation of GAGs in vascular cells therefore might be a fundamental process in the sense of a “gut–matrix axis” that precedes further pathophysiological changes in the cardiovascular tissue.

## 6. Materials and Methods

### 6.1. Cell Culture

Primary rat VSMCs were obtained from carotid arteries of male Wistar rats as described [61]. Together with ECs of the line EA.Hy926, the cells were grown in DMEM (Life Technologies, Hennigsdorf, Germany), which contained 10% FCS, 2 mM l-glutamine, 50 μg/mL streptomycin, and 50 U/mL penicillin. The cells were passaged ~80% confluence, 37 °C, and 5% CO_2_.

### 6.2. Rat Model of CKD

The 8–10-week-old male Wismar rats (Charles River, Écully, France) were fed with Adenin (0.3% Adenin, 1.2% Ca, 1.2% P, 20% Lactose, 1000IE Vitamin D, Altromin, Lage, Germany) for 7–10 weeks. Aortas were rapidly excised, fixed in formalin (10% *v*/*v*) for 48 h, followed by histological analysis.

### 6.3. Aortic Ring Culture

Thoracic aortas derived from healthy male Wistar rats were cleaned of connective tissue and cut into rings of 2 to 3 mm for ex vivo experiments with UTs. Rings were incubated with UTs for 7 d in DMEM at 37 °C. The UT concentrations used were 3.0 mM NaH_2_PO_4_ (Pi, final concentration), 50 µg/mL IS, 100 µM TMAO, 20 mM UREA, and 100 µg/mL pCS (Merck, Darmstadt, Germany). These concentrations correspond to mean plasma concentrations in uremic patients [62].

### 6.4. Histological Staining of Aortic Rings

Paraffin-embedded aortic rings were cut into 5 µm sections and mounted on SuperFrost Ultra Plus slides (Menzel GmbH, Braunschweig, Germany) prior to histological staining with alcian blue (pH 2.5), Movat pentachrome, and HALE.

Images of stained aortic rings were taken using a Carl Zeiss Imager A1 (Axio Vision 4.7.2) microscope, at ×20 magnification (Carl Zeiss, Oberkochen, Germany). The same optic characteristics were used for all samples.

Staining intensities were determined by two blinded colleagues using the Image J software (version 1.50i; National Institutes of Health, Bethesda, MA, USA) and are expressed in relative units.

### 6.5. Treatment of Cells with UTs

Primary VSMCs and ECs were treated for up to 7 d in 6-well or 12-well plates (NUNC, Roskilde Denmark) with the same concentrations as for the aortic rings.

### 6.6. PCR Measurements

TaqMan™ assays were used to determine relative gene expressions in aortic rings and cultured cells by qPCR. All applied TaqMan probes are listed in Table 1. The housekeeping gene ribosomal protein L19 (RPL19) was used to normalize the gene expressions.

### 6.7. Determination of sGAG Contents in Aortic Rings and Cells

Contents of sGAGs in aortic rings and cultured cells were determined using the Blyscan^TM^ Sulphated Glycosaminoglycan Assay (Biocolor, UK) as described in detail earlier [18]. Briefly, treated cells were digested with papain solution prior to the incubation with the Blyscan^TM^-dye reagent. Amounts of sGAGs were quantified photometrically and were normalized to the amounts of double-stranded DNA in the samples as described [63].

### 6.8. Determination of HA Contents in Cells

The effects of UTs on HA contents in the cells were measured as described in detail [19] using the HA QuantikineTM ELISA Kit (R&D Systems, Minneapolis, MN, USA). All sample lysates were analyzed in triplicates.

### 6.9. Western-Blot Analyses

Gel electrophoresis and western blots were determined as described earlier [19]. Table 2 lists the primary antibodies that were used.

### 6.10. Inhibition of AKT Signaling in Cultured Cells

PI3K/AKT signaling was blocked using 20 µM LY294002 (Ly29, Cell signaling) as described [19].

### 6.11. HS and CS/DS Disaccharide Analysis of Vascular Cells

Cell lysates were resuspended in 50 mM Tris / 10 mM CaCl_2_ pH 7.6 and disaccharide analyses were performed like described earlier [18].

### 6.12. Measurement of NF-κB Activation in Vascular Cells

Effects of UTs on NF-κB activity in ECs and VSMCs were determined using a luciferase reporter assay as described [64]. Briefly, the cells were transiently transfected in white 96-well plates (NUNC) with the reporter plasmids pGL4.32[luc2P/NF-ĸB-RE/Hygro] and pGL4.74[hRluc/TK] (Promega, Walldorf, Germany) using the HiPerFect Transfection Reagent (Qiagen, Hilden, Germany). A total of 24 h after transfection, the cells were treated as indicated for 24 h and analyzed using the Dual-Glo Luciferase Assay System (Promega) on a Victor3 microplate reader (Perkin Elmer, Waltham, MA, USA).

### 6.13. Statistics

Statistical analyses were performed using GraphPad Prism (GraphPad Software, Version 6.01, La Jolla, CA, USA). Data sets were tested for outliers and normal distribution. The in vitro data were analyzed as follows: two treatment groups were compared by using unpaired *t* tests. Two treatment groups with different variables were compared by 2-way ANOVA and Sidak’s multiple comparisons test. Comparisons of three groups were achieved by 1-way ANOVA and Tukey’s multiple comparisons test. The ex vivo-derived data were analyzed by Kruskal–Wallis test or 2-way ANOVA with Dunnett’s multiple comparisons test. The in vivo-derived aortas were analyzed by Mann–Whitney test. *p*-values of <0.05 were considered statistically significant.

## Figures and Tables

**Table 1:** Applied Taqman probes (ThermoFisher, Hennigsdorf, Germany) in the qPCR experiments.

Gene	Full Name	Assay-ID (Rat)	Assay-ID (Human)
B3GNT2	Beta-1,3-N-acetylglucosaminyltransferase 2	Rn02112835_s1	Hs01935859_s1
B4GALT1	Beta-1,4-galactosyltransferase 1	Rn01764643_m1	Hs00419232_g1
CHST1	Carbohydrate sulfotransferase 1	Rn01484520_m1	Hs04972213_s1
CHST15	Carbohydrate sulfotransferase 15	Rn00597859_m1	Hs01031067_m1
CHSY1	Chondroitin sulphate synthase 1	Rn01478125_m1	Hs00208704_m1
EXT1	Exostosin Glycosyltransferase 1	Rn00468764_m1	Hs00609162_m1
HAS1	Hyaluronan synthase 1	Rn01455687_g1	Hs00608272_m1
HEXA	Hexosaminidase subunit alpha	Rn01422539_m1	Hs00942655_m1
HEXB	Hexosaminidase subunit beta	Rn01493909_m1	Hs01077594_m1
RPL19	Ribosomal Protein L19	Rn00821265_g1	Hs02338565_gH
SULF1	Sulfatase 1	Rn00592734_m1	Hs00392834_m1
SULF2	Sulfatase 2	Rn01423347_m1	Hs01016480_m1
XYLT1	Xylosyltransferase 1	Rn01755138_m1	Hs00544498_m1
XYLT2	Xylosyltransferase 2	Rn00574186_m1	Hs01048792_m1

**Table 2:** Primary antibodies used for western-blot analyses.

Target	Dilution	Company	Product Number
AKT	1:1000	Cell signaling (Danvers, MA, USA)	#9272
p-AKT	1:1000	Cell signaling	#4056
B4GALT1	1:1000	ThermoFisher	#PA5-52744
HAS1	1:800	ThermoFisher	#PA5-95599
XYLT2	1:800	ThermoFisher	#PA5-29127
β-actin	1:1000	Sigma-Aldrich (Taufkirchen, Germany)	#A2228

## Data Availability

The original contributions presented in this study are included in the article. Further inquiries can be directed to the corresponding author(s).

## Abbreviations

5-UT	mixture of the five uremic toxins (indoxylsulphate, p-cresylsulphate, trimethylamine N-oxide, inorganic phosphate and urea)
CKD	chronic kidney disease
CS	chondroitin sulphate
ECs	endothelial cells
GAGs	glycosaminoglycans
HA	hyaluronic acid
HS	heparan sulphate
IS	indoxylsulphate
pCS	p-cresylsulphate
Pi	inorganic phosphate
sGAGs	sulphated glycosaminoglycans
TMAO	trimethylamine N-oxide
UTs	uremic toxins
VSMCs	vascular smooth muscle cells
